# Distinguishing EGFR mutant subtypes in stage IA non-small cell lung cancer using the presence status of ground glass opacity and final histologic classification: a systematic review and meta-analysis

**DOI:** 10.3389/fmed.2023.1268846

**Published:** 2023-12-06

**Authors:** Jianhao Qiu, Zheng Ma, Rongyang Li, Chenghao Qu, Kun Wang, Binyan Liu, Yu Tian, Hui Tian

**Affiliations:** ^1^Department of Thoracic Surgery, Qilu Hospital of Shandong University, Jinan, Shandong, China; ^2^Department of Breast Surgery, Qilu Hospital of Shandong University, Jinan, Shandong, China

**Keywords:** non-small cell lung cancer, ground glass opacity, histology, epidermal growth factor receptor, thoracic computed tomography, meta-analysis

## Abstract

**Background:**

The progression of early stage non-small cell lung cancer (NSCLC) is closely related to epidermal growth factor receptor (EGFR) mutation status. The purpose of this study was to systematically investigate the relationship between EGFR mutation status and demographic, imaging, and ultimately pathologic features in patients with NSCLC.

**Methods:**

A complete literature search was conducted using the PubMed, Web of Science, EMBASE, and Cochrane Library databases to discover articles published by May 15, 2023 that were eligible. The relationship between EGFR mutation status and specific demographic, imaging, and ultimately pathologic features in patients with NSCLC was evaluated using pooled odds ratios (ORs) and their 95% confidence intervals (CIs). The standardized mean difference (SMD) with 95% CIs was the appropriate statistic to summarize standard deviations (SDs) means for continuous variables.

**Results:**

A total of 9 studies with 1789 patients were included in this analysis. The final findings suggested that patients with a greater age, female gender, and non-smoking status would have a relatively higher incidence of EGFR mutations. Additionally, the risk of EGFR mutations increased with larger tumor diameter, tumor imaging presentation of mixed ground glass opacity (mGGO), and tumor pathological findings of minimally invasive adenocarcinoma (MIA) or invasive adenocarcinoma (IAC). Significantly, malignancies presenting as MIA are more likely to contain L858R point mutations (OR = 1.80; 95% CI: 1.04–3.13; *p* = 0.04) rather than exon 19 deletions (OR = 1.81; 95% CI: 0.95–3.44; *p* = 0.07).

**Conclusion:**

This meta-analysis showed that imaging parameters and histological classifications of pulmonary nodules may be able to predict stage IA NSCLC genetic changes.

## Introduction

Lung cancer has surpassed colonic and prostate cancers as a leading cause of cancer-related deaths globally, due to improvements in early detection and lowered average ages at diagnosis (1). Authoritative research had shown that the development of non-small cell lung cancer (NSCLC) may be influenced by the epidermal growth factor receptor (EGFR) (2). Among the frequent EGFR gene mutations, the L858R point mutations in exon 21 accounts for 40% and the exon 19 deletion mutations (19del) accounts for 45% (3, 4). Both variants have been named sensitive mutations. Additional EGFR mutations (G719X, S768I, L861Q, etc.) are classified as rare mutations. Since treatment with epidermal growth factor receptor-tyrosine kinase inhibitors (EGFR-TKI) has been demonstrated to dramatically enhance the survival of patients with NSCLC, the identification of EGFR mutations has become a crucial part of NSCLC treatment, particularly for lung adenocarcinoma (LUAD) (5–7).

Currently, EGFR-related studies are focusing on the link between EGFR mutations and NSCLC; nevertheless, this topic is fraught with controversy. The findings of studies by Yotsukura et al. Zhang and et al., which suggest that EGFR mutations are early genomic events in LUAD (8, 9), while Zhu et al. suggest that the frequency of EGFR mutations is not significantly different in minimally invasive adenocarcinoma (MIA) and invasive adenocarcinoma (IAC) (10). Also of great interest is whether or not there is a correlation between the imaging characteristics of LUAD and the frequency with which it undergoes EGFR mutations. Wei et al. found no link between EGFR and ground glass opacity (GGO) development, but Ortiz et al. found an increased probability of EGFR mutations when GGO was present in pulmonary nodules (11, 12). As a result, we aimed to perform a systematic review and meta-analysis of the literature to learn more about the correlation between EGFR mutations and the percentage of ground glass opacity and final histological classification in people with NSCLC.

## Materials and methods

### Protocol and ethics statement

This systematic review and meta-analysis report was conducted in accordance with the PRISMA (Preferred Reporting Items for Systematic Reviews and Meta-Analyses) and MOOSE (Meta-Analysis of Observational Studies in Epidemiology) guidelines and statements (13, 14). This protocol for a systematic review and meta-analysis has registered on the INPLASY website^1^ with the registration number INPLASY202320043.

### Databases and search strategy

The literature review was conducted using four online databases: Pubmed, EMBASE, Cochrane Library and Web of Science until 15 May 2023. The medical keywords included in the search strategy were “lung neoplasms,” “carcinoma, non-small-cell lung,” “lung adenocarcinoma,” “carcinoma, squamous cell,” “ground glass opacity,” and “epidermal growth factor receptor.” The MeSH terminology database of the National Center for Biotechnology Information (NCBI) was queried to identify all potential expressions of these terms. For each valid combination of the two Boolean operators (“AND” and “OR”), the keywords and free words were employed. Supplementary Table S1 describes in detail the search strategy for all databases. Two authors (Jianhao Qiu and Zheng Ma) independently evaluated and cross-checked each article. In addition, we manually reviewed the reference lists of eliminated publications to ensure that we did not overlook viable, non-repetitive investigations. Any disagreements between reviewers were resolved through conversation.

### Study selection and criteria

The following were the selection criteria: (1) involved adult patients who underwent pneumonectomy or histological analysis of the lung (puncture biopsy or bronchoscopic biopsy); (2) all patients were tested for EGFR mutations (detection methods included polymerase chain reaction, immunohistochemistry, and DNA sequencing); (3) involved a group of patients with EGFR mutant-positive results (mutations sites included all mutant subtypes); (4) involved a group of patients with EGFR mutant-negative results as controls; (5) at least one of the pertinent results of interest was reported (see below); (6) written in English.

The criteria for exclusion were as follows: (1) ineligible article types such as case reports, reviews, conference abstracts, non-comparative studies; (2) no outcome of interest; (3) insufficient or missing data for analysis; (4) written in a language other than English; (5) non-human participants.

### Endpoints and outcome measures

The primary outcome of interest was the relationship between EGFR gene mutations and the percentage of GGO and final histological classification in patients’ tumors. Other relevant indicators included mainly demographic characteristics such as age, gender, and smoking history. In addition an analysis of the imaging size of the tumor was made. The GGO was defined as ground glass dense nodules with visible internal vessels and bronchi. If the GGO is composed of ground glass opacity only, it is defined as pure ground glass opacity (pGGO). If the GGO is a combination of ground glass opacity and solid components, it is defined as mixed ground glass opacity (mGGO). The tumor imaging size was defined as the maximum diameter of the tumor on the axial image of a certain slice on the lung window of the thoracic computed tomography (CT).

### Data extraction

The following data were extracted from each study: (1) publication data: authors, year and country of publication; (2) experimental data: study design and period, method of EGFR gene testing and test range; (3) demographic data: number of cases, age, gender and smoking history of included samples; (4) outcome data: tumor size, proportion of ground glass components, imaging characteristics and pathological classification. Two writers (Jianhao Qiu and Zheng Ma) independently examined the relevant studies and retrieved the necessary information to fill out the predesigned forms. All disputes were settled by consensus. We did not communicate with the authors about unpublished data.

### Quality of evidence

In this systematic review and meta-analysis, the quality of case–control studies was assessed using the Newcastle-Ottawa Quality Assessment Scale (NOS) (15). Studies with a score of 6 or higher were considered eligible for further meta-analysis. The quality of each study was independently assessed by two authors (Jianhao Qiu and Zheng Ma). Any differences in quality assessment were resolved by consensus.

### Statistical analysis

All statistical analyses were conducted using the Review Manager software (RevMan version 5.3; The Nordic Cochrane Center, The Cochrane Collaboration, 2014) and the STATA 16 software package (StataCorp LLC, College Station, TX).

We calculated 95% confidence interval (CI) and odds ratio (OR) to summarize the relationship between dichotomous data and EGFR gene mutations. The standardized mean difference (SMD) with 95% CIs was the appropriate statistic to summarize standard deviations (SDs) means for continuous variables. If SDs were not supplied, we would not have included the data in the quantitative synthesis since, according to Cochrane Collaboration criteria, extrapolation of SDs is only applicable to trials with large sample sizes and normally distributed outcomes (16).

This systematic review and meta-analysis used the Cochrane Q test and the I^2^ statistic to quantify the degree of heterogeneity, with an I^2^ greater than 50% considered to be substantial (17). A two-tailed *p* < 0.05 was considered statistically significant. If the I^2^ test value was <50%, we used a fixed-effect model; nevertheless, we used a random-effect model if the I^2^ test value was >50% (18). Egger’s test was used to detect possible publication bias, and the presence of significant publication bias was determined if *p* < 0.05 for Egger’s test (19).

The stability of the pooled estimates was further examined using a sensitivity analysis, in which the effect of each study on the overall estimate could be tested by sequentially omitting individual studies.

## Results

### Literature search

**Figure 1:**
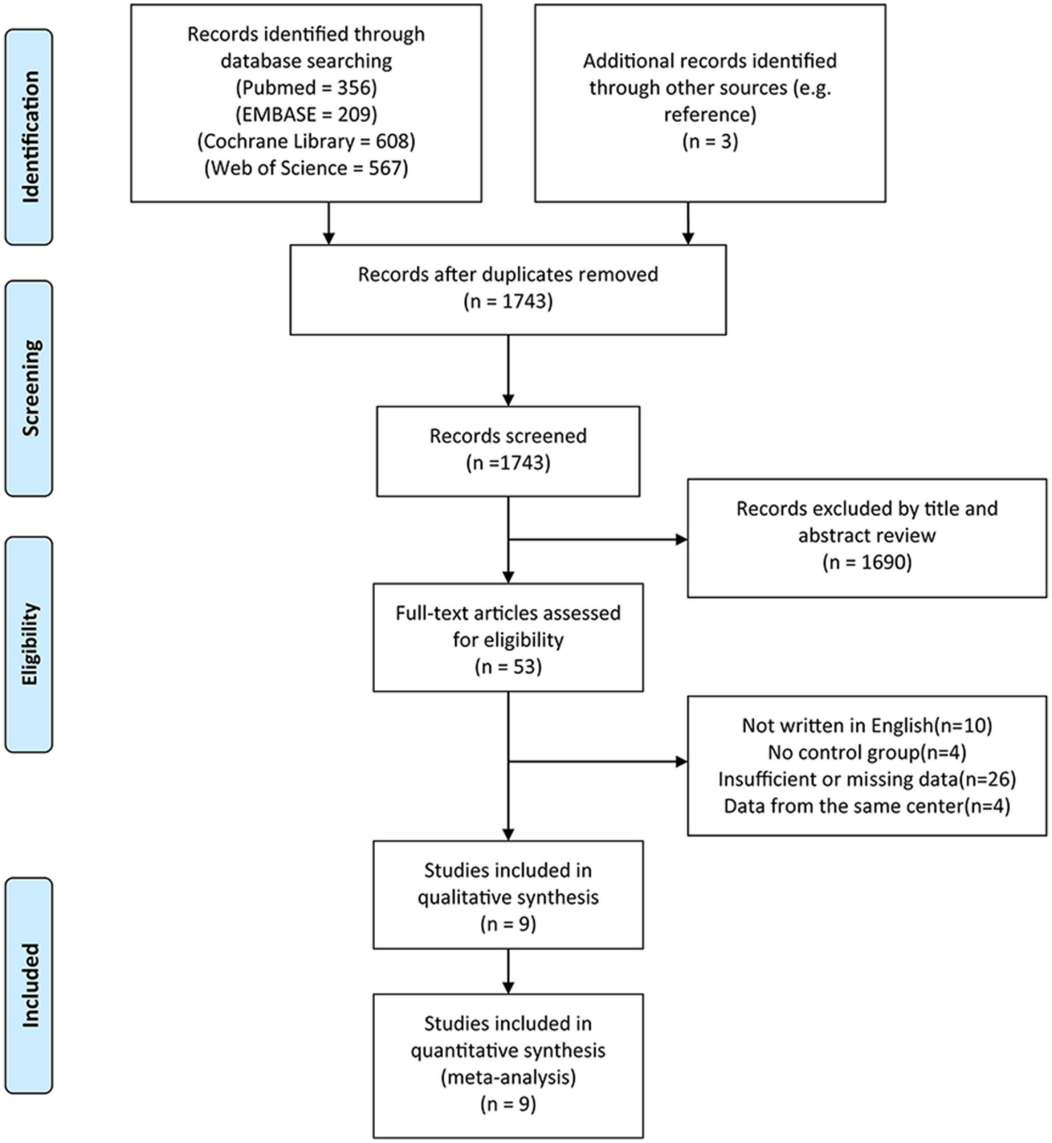
PRISMA flow diagram of literature retrieval. PRISMA, Preferred Reporting Items for Systematic Reviews and Meta-Analyses.

A flow diagram outlining the search process showed Figure 1. A total of 1,740 potential articles were identified, including 356 PubMed citations, 209 Embase citations, 608 Cochrane Library citations, and 567 Web of Science citations. In addition, a manual literature search of the reference list yielded three relevant studies. A total of 9 articles were finally included in this meta-analysis after checking for duplicates and screening titles, abstracts, and full texts (20–28).

### Characteristics of the included studies

The baseline features of each research that met the inclusion criteria were summarized in Table 1, and relevant demographic, imaging, and histopathological outcomes were presented in Tables 2, 3. The 9 retrospective studies included in this meta-analysis were conducted between 2009 and 2023 in three different countries, with sample sizes ranging from 24 to 429 patients. A total of 1789 patients were finally included in this meta-analysis. The majority of patients were from China (*n* = 1,232; 68.87%), followed by 453 patients from Korea (25.32%), only one study was from Japan (*n* = 104; 5.81%). Regarding the EGFR mutations rate, the study with the highest mutations rate was from Japan (64.42%) (23). The study with the lowest mutations rate was from China (31.63%) (28). Almost all studies tested common mutation sites in the EGFR gene to varying degrees (e.g., L838R, 19del, etc.), and only one study from Korea did not specify the detailed sites of the mutations (25). The patients in the remaining 8 included literatures were all confirmed as having LUAD by postoperative pathology, and only one included literature had a postoperative pathological diagnosis of NSCLC (25).

**Table 1:** Baseline characteristics and methodological assessment of included studies.

Study (year)	Country	Period	Study design	Sample size	Stage	Pathology	Genetic testing method	EGFR mutation test	EGFR mutation rate
Chung et al. (20)	Korea	2003–2009	Retrospective	24	0-IA	LUAD	PCR	Exons 18–21	41.07%
Kobayashi et al. (23)	Japan	2012–2014	Retrospective	104	0-IA	LUAD	RT-PCR	Exons 19–21	64.42%
Wang et al. (27)	China	2011–2014	Retrospective	207	0-IA	LUAD	PCR	Exons 19, 21	35.27%
Dai et al. (21)	China	2013–2014	Retrospective	204	0-IA	LUAD	qPCR	Exons 18–21	53.43%
Lu et al. (24)	China	2013–2015	Retrospective	156	0-IA	LUAD	qPCR	Exons 18, 19, 21	48.08%
Wang et al. (26)	China	2014–2017	Retrospective	309	0-IA	LUAD	PCR	Exons 18–21	52.75%
Zhu et al. (28)	China	2011–2017	Retrospective	98	0-IA	LUAD	qPCR and IHC	Exons 18–21	31.63%
Tsai et al. (25)	Korea	2009–2014	Retrospective	429	0-IA	NSCLC	PCR	NR	48.25%
Ji et al. (22)	China	2021.01–06	Retrospective	258	0-IA	LUAD	PCR	Exons 18–21	38.76%

NR, not reported; EGFR, epidermal growth factor receptor; LUAD, lung adenocarcinoma; NSCLC, non-small cell lung cancer; PCR, polymerase chain reaction; RT-PCR, reverse transcription-polymerase chain reaction; qPCR, quantitative polymerase chain reaction; IHC, immunohistochemistry.

**Table 2:** Detailed demographic and preoperative CT characteristic data of the included studies.

Study (year)	Age (years)		Gender, female (%)		Smoking history (%)		Size (cm)		mGGO (%)	
	Wild	Mut	Wild	Mut	Wild	Mut	Wild	Mut	Wild	Mut
Chung et al. (20)	55.6 ± 10.8	59.6 ± 14.6	1 (20.0)	6 (42.9)	3 (60.0)	6 (42.9)	1.06 ± 0.79	1.23 ± 0.82	13 (48.1)	10 (55.6)
Kobayashi et al. (23)	NR	NR	23 (62.2)	39 (58.2)	19 (51.4)	27 (40.3)	NR	NR	28 (75.7)	54 (80.6)
Wang et al. (27)	NR	NR	34 (25.4)	45 (61.6)	87 (64.9)	26 (35.6)	NR	NR	39 (38.6)	21 (52.5)
Dai et al. (21)	57.8 ± 10.4	58.6 ± 9.5	46 (48.4)	74 (67.9)	54 (56.8)	32 (29.4)	1.26 ± 0.51	1.46 ± 0.49	NR	NR
Lu et al. (24)	NR	NR	37 (45.7)	51 (68.0)	26 (32.1)	12 (16.0)	1.31 ± 0.40	1.34 ± 0.36	75 (92.6)	67 (89.3)
Wang et al. (26)	50 ± 14	51 ± 13	54 (37.0)	90 (55.2)	90 (61.6)	66 (40.5)	NR	NR	61 (41.8)	73 (44.8)
Zhu et al. (28)	62.7 ± 15.6	66.6 ± 14.2	37 (55.2)	19 (61.3)	24 (35.8)	8 (25.8)	1.437 ± 0.630	1.719 ± 0.679	46 (68.7)	24 (77.4)
Tsai et al. (25)	60.3 ± 11.1	62.7 ± 9.0	150 (67.6)	153 (73.9)	51 (23.0)	35 (16.9)	1.17 ± 0.62	1.48 ± 0.66	NR	NR
Ji et al. (22)	50.7 ± 10.2	56.3 ± 10.6	97 (61.4)	55 (55.0)	37 (23.4)	26 (26.0)	NR	NR	37 (30.8)	44 (50.6)

CT, computed tomography; mGGO, mix ground glass opacity; Mut, mutation; NR, not reported.

**Table 3:** Detailed tumor histology and EGFR mutation subtype data of the included studies.

Study (year)	IAC (%)				MIA (%)				PGL (%)			
	Wild	Mut	19del	21 (L858R)	Wild	Mut	19del	21 (L858R)	Wild	Mut	19del	21 (L858R)
Chung et al. (20)	9 (33.3)	7 (36.8)	5 (26.3)	1 (5.3)	NR	NR	NR	NR	18 (66.7)	12 (63.2)	8 (42.1)	4 (21.1)
Kobayashi et al. (23)	16 (43.2)	39 (58.2)	NR	NR	9 (24.3)	18 (26.9)	NR	NR	12 (32.4)	10 (14.9)	NR	NR
Wang et al. (27)	57 (42.5)	44 (60.3)	25 (34.2)	19 (26.0)	44 (32.8)	18 (24.7)	10 (13.7)	8 (11.0)	33 (24.6)	11 (15.1)	4 (5.5)	7 (9.6)
Dai et al. (21)	38 (40.0)	73 (67.0)	25 (22.9)	45 (41.3)	26 (27.4)	24 (22.0)	12 (11.0)	10 (9.2)	31 (32.6)	12 (11.0)	6 (5.5)	6 (5.5)
Lu et al. (24)	55 (67.9)	45 (60.0)	NR	NR	7 (8.6)	10 (13.3)	NR	NR	19 (23.5)	20 (26.7)	NR	NR
Wang et al. (26)	NR	NR	NR	NR	NR	NR	NR	NR	NR	NR	NR	NR
Zhu et al. (28)	58 (86.6)	26 (83.9)	NR	NR	NR	NR	NR	NR	9 (13.4)	5 (16.1)	NR	NR
Tsai et al. (25)	NR	NR	NR	NR	NR	NR	NR	NR	NR	NR	NR	NR
Ji et al. (22)	33 (20.9)	41 (41.0)	14 (14.0)	24 (24.0)	34 (21.5)	25 (25.0)	6 (6.0)	17 (17.0)	91 (57.6)	34 (34.0)	12 (12.0)	18 (18.0)

EGFR, epidermal growth factor receptor; IAC, invasive adenocarcinoma; MIA, minimally invasive adenocarcinoma; PGL, precursor glandular lesions; Mut, mutation; 19del, 19 deletion mutations; 21 (L858R), the L858R point mutations in exon 21 accounts; NR, not reported.

### Quality assessment

The quality assessment of the included studies is shown in Table 4. All 9 included retrospective case–control studies had a NOS score greater than 6, which is an indication that they were all of acceptable quality and no other risk of bias was found.

**Table 4:** Detailed quality assessment of included studies.

Items of NOS	Included studies								
	Chung et al. (20)	Kobayashi et al. (23)	Wang et al. (27)	Dai et al. (21)	Lu et al. (24)	Wang et al. (26)	Zhu et al. (10)	Tsai et al. (25)	Ji et al. (22)
Selection									
Representativeness of the exposed cohort	★	★	★	★	★	★	★	★	★
Selection of the non-exposed cohort		★	★		★	★			★
Ascertainment of exposure	★	★	★	★	★	★	★	★	★
Demonstration that outcome of interest was not present at start of study	★	★	★	★	★	★	★	★	★
Comparability									
Comparability of cohorts on basis of the design or analysis	★★	★★	★★	★★	★	★	★	★	★★
Outcome									
Assessment of outcome	★	★	★	★	★	★	★	★	★
Follow-up long enough for outcomes to occur	★	★	★	★	★	★	★	★	★
Adequacy of follow up of cohorts	★	★	★	★	★	★	★	★	★
Total	8	9	9	8	8	8	7	7	9

A study can be awarded a maximum of one star for each numbered item within the Selection and Outcome categories. A maximum of two stars can be given for Comparability. Study rates ≥ 6 is eligible for further analysis. NOS, Newcastle-Ottawa Scale.

#### Older age and EGFR mutations

**Figure 2:**
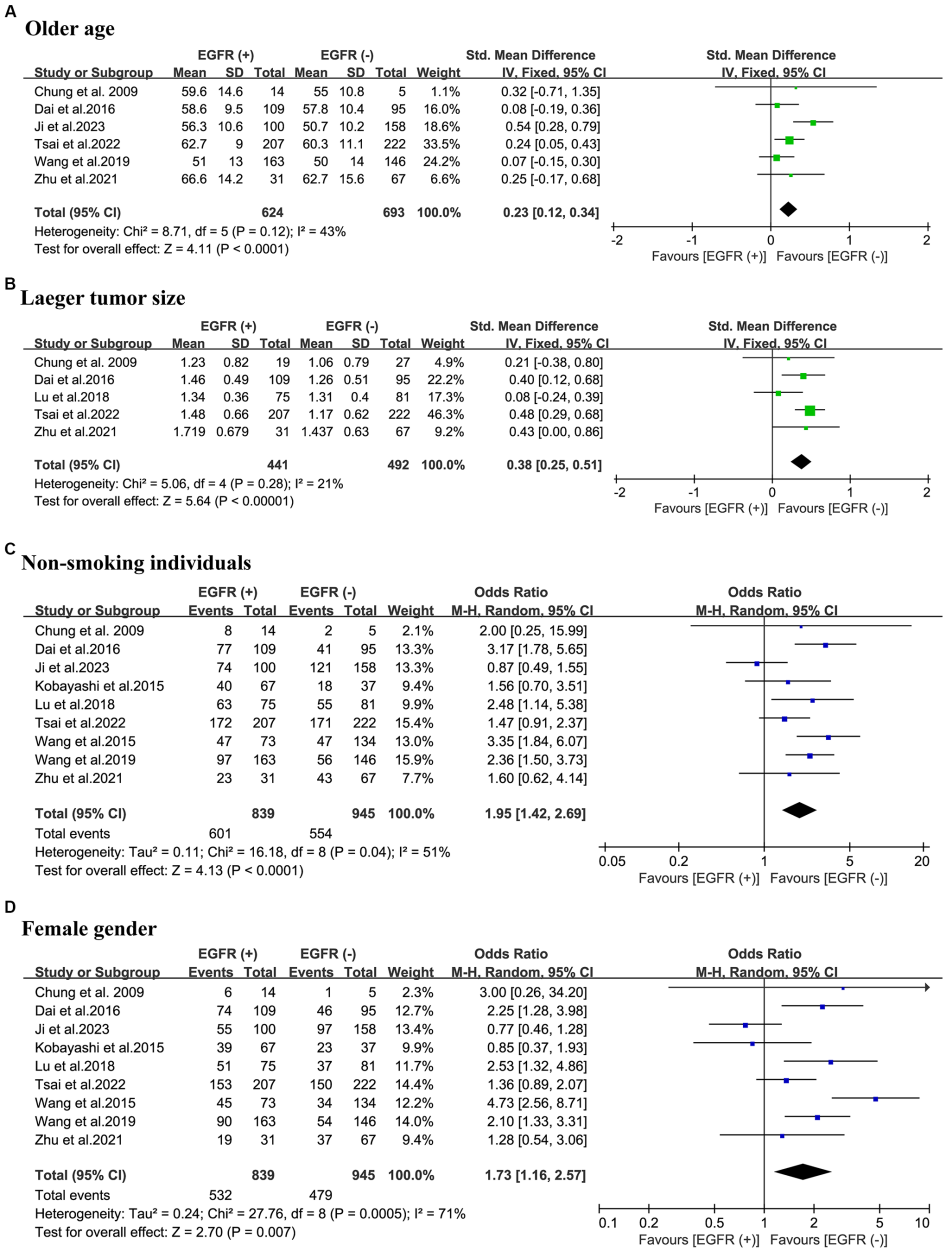
Meta-analysis of demographic and partial thoracic CT information of patients with NSCLC between the EGFR mutant-positive and mutant-negative groups. **(A)** Old age; **(B)** larger tumor size; **(C)** non-smoking individuals; **(D)** female gender. EGFR (+), EGFR mutations were positive; EGFR (−), EGFR mutations were negative; CT, computed tomography; NSCLC, non-small cell lung cancer; EGFR, epidermal growth factor receptor; OR, odds ratio; CI, confidence interval.

A total of 6 studies were included, reporting the age of 1,317 patients in relation to the EGFR mutations status of their tumors. The results of the meta-analysis showed that patients in the EGFR mutant-positive group were older compared to the EGFR mutant-negative group (SMD = 0.23; 95% CI: 0.12–0.34; *p* < 0.001) and a relatively low heterogeneity (I^2^ = 43%; *p* = 0.12). Using Egger’s test, no publication bias was discovered (*p* = 0.843) (Figure 2A).

#### Larger tumor size and EGFR mutations

The relationship between tumor size and EGFR mutations was evaluated in a total of 933 patients from 5 studies. The meta-analysis revealed that patients with EGFR mutations exhibited larger tumor sizes (SMD = 0.38; 95% CI: 0.25–0.51; *p* < 0.001) and a relatively low heterogeneity (I^2^ = 21%; *p* = 0.28). The Egger’s test did not reveal any publication bias (*p* = 0.382). (Figure 2B).

### Non-smoking individuals and EGFR mutations

The association between patients’ smoking history and EGFR mutations was examined by pooling of 1784 patients from 9 studies in 3 countries. This meta-analysis indicated that the occurrence rate of non-smoking individuals in the EGFR mutant-positive group was significantly higher than in the EGFR mutant-negative group (OR = 1.95; 95% CI: 1.42–2.69; *p* < 0.001). There was a high-degree of heterogeneity (I^2^ = 51%; *p* = 0.04). The Egger’s test revealed no evidence of publication bias (*p* = 0.964) (Figure 2C).

### Female gender and EGFR mutations

To evaluate the association between patient gender and EGFR mutations, 1784 patients from 9 different studies were pooled. The meta-analysis elucidates the higher proportion of the female population in the EGFR mutant-positive group compared to the EGFR mutant-negative group (OR = 1.73; 95% CI: 1.16–2.57; *p* = 0.007) with a considerable heterogeneity (I^2^ = 71.0%; p < 0.001). No publication bias was found using Egger’s test (*p* = 0.803) (Figure 2D).

### mGGO and EGFR mutations

**Figure 3:**
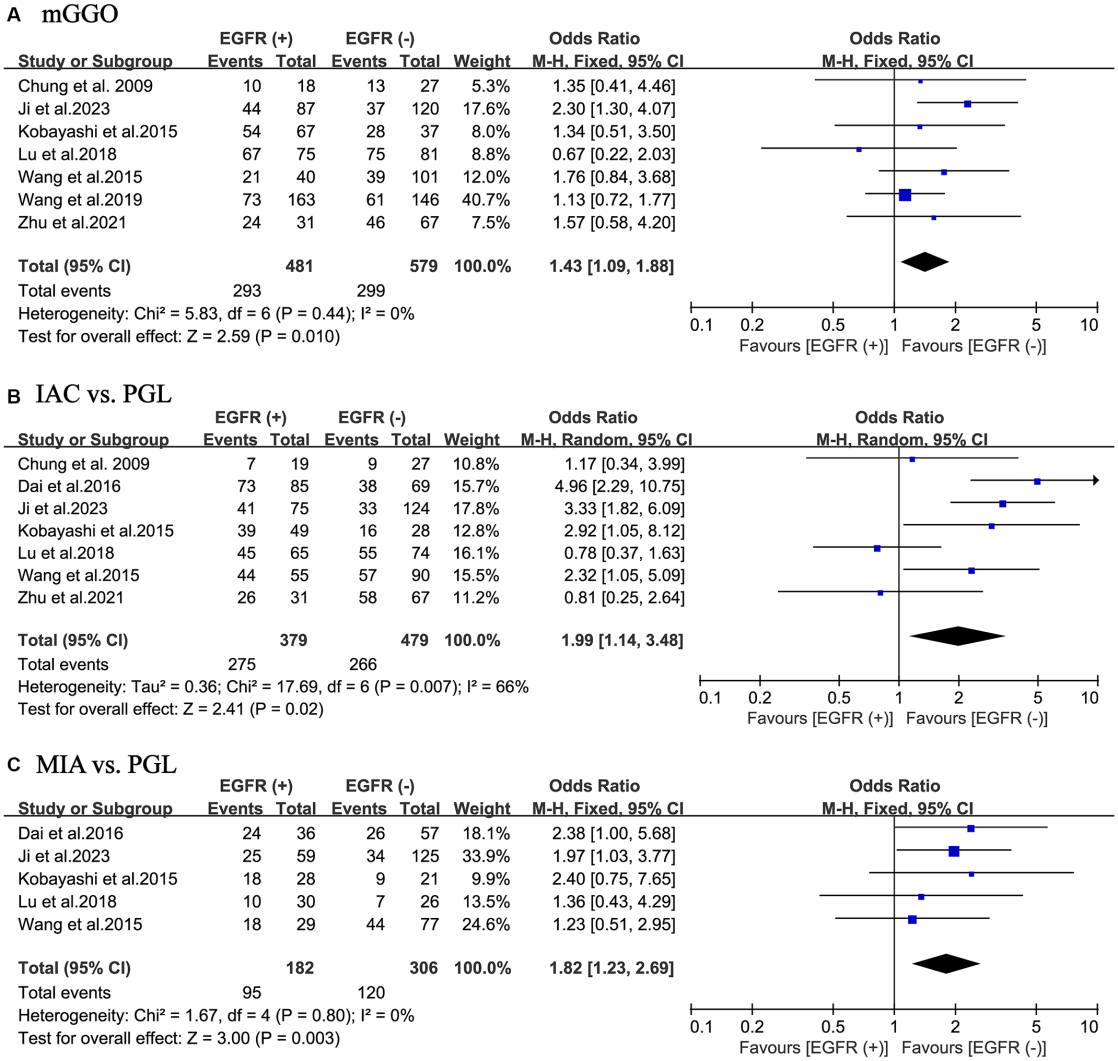
Meta-analysis of partial thoracic CT information and pathological outcomes of patients with NSCLC between the EGFR mutant-positive and mutant-negative groups. **(A)** mGGO; **(B)** IAC vs. PGL; **(C)** MIA vs. PGL. EGFR (+), EGFR mutations were positive; EGFR (−), EGFR mutations were negative; CT, computed tomography; NSCLC, non-small cell lung cancer; EGFR, epidermal growth factor receptor; mGGO, mixed ground glass opacity; IAC, invasive adenocarcinoma; MIA, minimally invasive adenocarcinoma; PGL, precursor glandular lesions; OR, odds ratio; CI, confidence interval.

Overall, 1,060 patients from 7 studies were pooled to investigate the relationship between the percentage of GGO component in their tumors and EGFR mutations. The results of the meta-analysis indicated that in the EGFR mutant-positive group compared to the EGFR mutant-negative group, there was a statistically significant increase in the incidence of pulmonary nodules as mGGO (OR = 1.43; 95% CI: 1.09–1.88; *p* = 0.010) with a slight heterogeneity (I^2^ = 0%; *p* = 0.44). No publication bias was found using Egger’s test (*p* = 0.776) (Figure 3A).

### Histology and EGFR mutations

In total, the ultimate histological outcome of 858 patients from 7 included studies was evaluated for EGFR mutations. As shown in Figures 3B,C, the incidence of EGFR mutations is relatively high in either IAC (OR = 1.99; 95% CI: 1.14–3.48; *p* = 0.020) or MIA (OR = 1.82; 95% CI: 1.23–2.69; *p* = 0.003) relative to precursor glandular lesions (PGL). There was a considerable heterogeneity in study of IAC (I^2^ = 66%; *p* = 0.007), whereas there was a slight heterogeneity in study of MIA (I^2^ = 0%; *p* = 0.80). No publication bias was found using Egger’s test (*p* = 0.366 for IAC; *p* = 0.813 for MIA).

**Figure 4:**
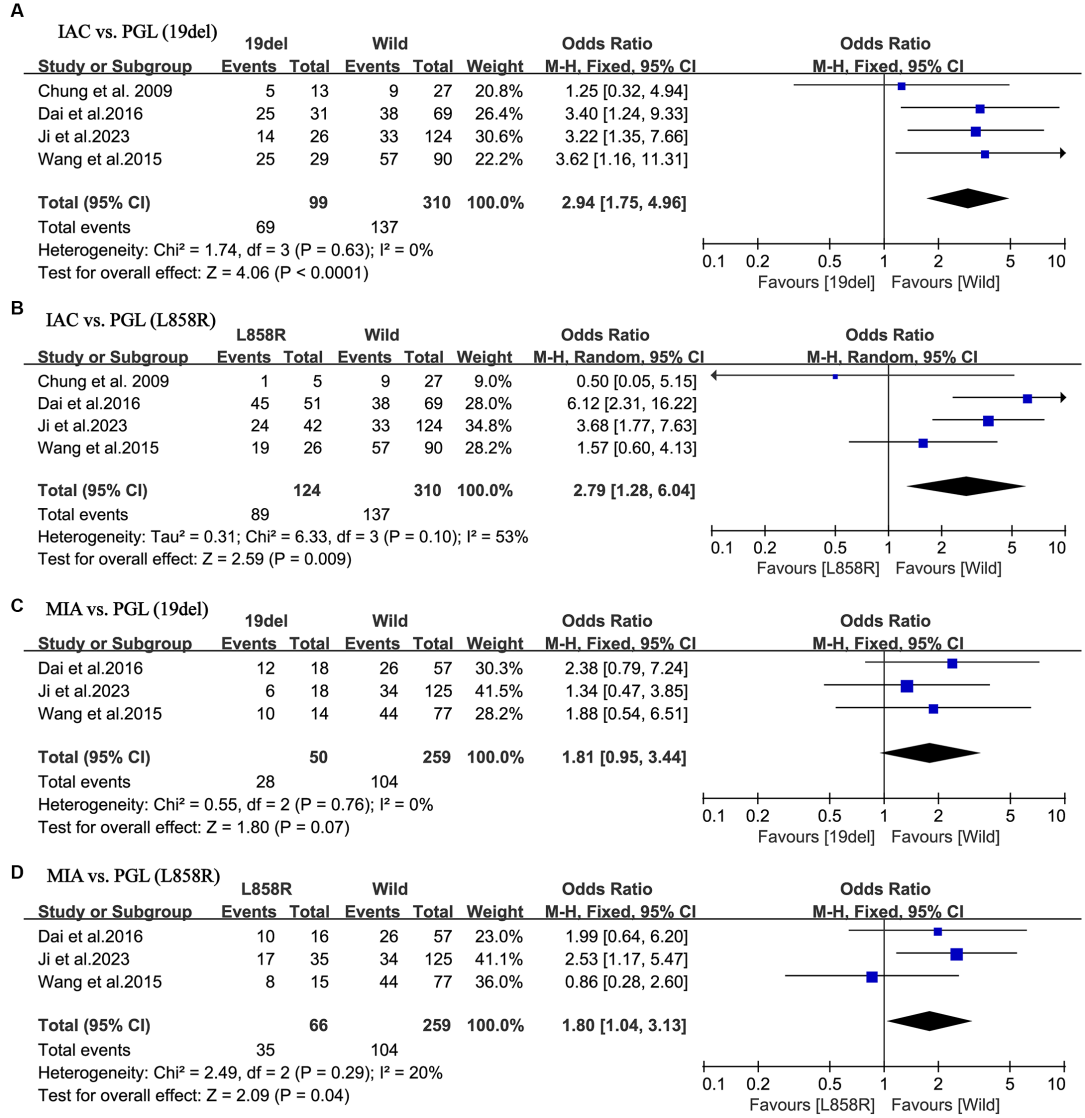
Meta-analysis of the pathological outcomes of patients with NSCLC between the EGFR mutant subtype groups and wild groups. **(A)** IAC vs. PGL (19del); **(B)** IAC vs. PGL (L858R); **(C)** MIA vs. PGL (19del); **(D)** MIA vs. PGL (L858R). NSCLC, non-small cell lung cancer; EGFR, epidermal growth factor receptor; IAC, invasive adenocarcinoma; MIA, minimally invasive adenocarcinoma; PGL, precursor glandular lesions; 19del, 19 deletion mutations; L858R, the L858R point mutations in exon 21 accounts; OR, odds ratio; CI, confidence interval.

A more thorough analysis was done to investigate into the relationship between EGFR mutant subgroups and tumor histology. As illustrated in Figures 4A,B, there was a higher incidence of IAC in both the exon 19 deletion group (OR = 2.94; 95% CI: 1.95–4.96; *p* < 0.001) and in the L858R point mutation group (OR = 2.79; 95% CI: 1.28–6.04; *p* = 0.009) compared to the negative group. There was minor heterogeneity in study of the exon 19 deletion (I^2^ = 0%; *p* = 0.63), whereas there was a considerable heterogeneity in study of the L858R point mutation (I^2^ = 66%; *p* = 0.007). No publication bias was detected in either of the two studies using Egger’s test (*p* = 0.270 for IAC; *p* = 0.378 for MIA).

Nevertheless, further studies on MIA demonstrated a different result. As shown in Figures 4C,D, MIA had a higher incidence in the L858R point mutation group, compared to the negative group (OR = 1.80; 95% CI: 1.04–3.13; *p* = 0.04). In contrast, in the exon 19 deletion group, there was no statistical difference compared to the negative group (OR = 1.81; 95% CI: 0.95–3.44; *p* = 0.07). Both groups of studies had low heterogeneity (I^2^ = 0%, *p* = 0.76 for the exon 19 deletion group; I^2^ = 20%, *p* = 0.29 for the L858R point mutation group). No publication bias was detected in either of the two studies using Egger’s test (*p* = 0.733 for IAC; *p* = 0.492 for MIA).

### Sensitivity analysis

We performed sensitivity analyses by sequentially omitting individual studies. As shown in Supplementary Figures S1–S3, none of the pooled ORs for the remaining studies in the analysis based on each component were outside the range of estimates. In addition, there were no significant differences between the corrected pooled estimates and the original pooled estimates. The reliability of our meta-analysis was thus validated.

## Discussion

A growing trend today is the identification of EGFR mutations in patients undergoing early diagnosis and therapy for NSCLC (29, 30). Although the conclusions of these investigations are still up for debate, prior researches have shown a connection between EGFR mutations and the imaging presentation of NSCLC and the ultimate histology of NSCLC. In order to draw more convincing conclusions, we conducted a systematic review and meta-analysis of 9 retrospective studies to examine the relationship between the clinical, imaging and histology characteristics of NSCLC patients and the frequency of EGFR mutations. This meta-analysis study discovered that age, tumor size, female gender, non-smoking individuals, pulmonary nodules presenting as mGGO, pulmonary nodules with histological types of MIA and IAC were significant factors influencing EGFR mutations. And further analysis confirmed that in the L858R point mutation group, there was a higher incidence of pulmonary nodules with histological type IAC or MIA. In contrast, in the exon 19 mutation group, only pulmonary nodules with IAC had a higher incidence, while pulmonary nodules with MIA were not statistically significant.

In this study, we discovered that patients who were substantially older had a higher risk of developing EGFR mutations than patients who were younger (SMD = 0.23; 95% CI: 0.12–0.34; *p* < 0.001). Multiple studies have shown that the risk of EGFR mutations increases with age in NSCLC patients (31, 32). Despite adjusting for patient histology, smoking status, and pathological staging, one study found an independent statistical difference between EGFR mutations and the age of the patient at diagnosis (33). In response to this conclusion, we suggest that those who are older have an increased risk of developing mutations due to changes in their own hormone levels, diminished nucleotide repair capacity, and increased exposure to carcinogenic stimuli from the environment (34–37). To validate this conclusion, additional large-scale gene sequencing with population representation is required.

The individual’s smoking history is also a significant determinant in EGFR mutations. This meta-analysis indicated that individuals without a history of smoking are more likely to have EGFR mutations (OR = 1.95; 95% CI: 1.42–2.69; *p* < 0.001). However, this analysis showed relatively high heterogeneity (I^2^ = 51%; *p* = 0.04). This may be attributable to variations in the statistical criteria for smoking history among the included studies. For example, the study by Wang et al. and Dai et al. defined patients who had quit smoking ≥1 year prior to the surgery were defined as former smokers (21, 27), whereas the remaining included studies did not make this distinction. In addition, we would have liked to use the smoking index, was defined as the number of cigarettes smoked per day multiplied by the number of years smoked, as a statistical measure of the extent of smoking among patients in this analysis to give more credibility. This statistical method was not utilized in any of the included studies, which prevented us from realizing our design. This can be explored further in a subsequent investigation.

Furthermore, the study revealed that the proportion of female patients in the EGFR mutant-positive group was significantly higher than in the EGFR mutant-negative group (OR = 1.73; 95% CI: 1.16–2.57; *p* = 0.007), meaning that women are more likely to develop EGFR mutations. Based on the findings of this study, we suggest that the estrogen level in females affects the incidence of EGFR mutations. Multiple studies have verified a positive correlation between estrogen receptors and EGFR mutations (38, 39). Additionally, the study by Mazières et al. confirmed that the expression of estrogen receptors was greater in non-smoking women than in smokers (40). This precisely explains why women who do not smoke have a higher lung cancer incidence rate. Chen et al. and Linardou et al. revealed a considerably greater probability of EGFR mutations in women in Asia, notably in East Asia (41, 42). The fact that each of the 9 studies we included was from East Asia further supports the reliability of our analysis.

For determining whether the tumor has an EGFR mutation, it’s also crucial to consider the size of the tumor and the percentage of GGO on the thoracic CT. The results of this meta-analysis revealed that the incidence of EGFR mutations was greater in pulmonary nodules exhibiting mGGO compared to those exhibiting pGGO (OR = 1.43; 95% CI: 1.09–1.88; *p* = 0.010). And the incidence of EGFR mutation was positively correlated with the imaging size of the tumor (SMD = 0.38; 95% CI: 0.25–0.51; *p* < 0.001). A study by Cai et al. in 2023 showed that pGGO and mGGO reflect the pathological development and genetic alterations of pulmonary nodules (43). This study suggests that the solid component of pGGO emerged and that the proportion of solid components progressively increased - manifesting as mGGO, which indicates progression of the tumor (i.e., pathologic findings of MIA or IAC) and an increase in the rate of EGFR mutations. Li et al. found that larger diameter tumors did reveal higher frequency and types of mutations in addition to EGFR mutations, such as ALK rearrangements, TP53 mutations, etc., in postoperative genetic testing (44).

EGFR mutations were strongly related with the histological type of the patient’s tumor. The results of the analysis indicate that the ultimate pathological outcomes of the tumors, whether MIA (OR = 1.82; 95% CI: 1.23–2.69; *p* = 0.003) or IAC (OR = 1.99; 95% CI: 1.14–3.48; *p* = 0.020), have a higher incidence of EGFR mutations than PGL. However, the meta-analysis of IAC showed a relatively high heterogeneity (I^2^ = 66%; *p* = 0.007), which might be due to subjective bias in the interpretation of IAC criteria by different institutions and different pathologists in the included studies. Several studies have shown that EGFR amplification is essential for the progression of AIS to MIA and even IAC (9, 45). Moreover, the risk of developing secondary primary lung cancer is marginally increased in MIA patients with EGFR mutations (8).

After conducting additional analysis for various subtypes of mutants, we arrived at contrasting conclusions. For tumors with pathological type IAC, both exon 19 deletion and L858R point mutation have a high incidence of mutations (OR = 2.94, 95% CI: 1.95–4.96, *p* < 0.001 for exon 19 deletion; OR = 2.79, 95% CI: 1.28–6.04, *p* = 0.009 for L858R point mutation). In contrast, tumors exhibiting MIA maintained a high mutation incidence only for the L858R point mutation (OR = 1.80; 95% CI: 1.04–3.13; *p* = 0.04), while losing statistical significance for the exon 19 deletion (OR = 1.81; 95% CI: 0.95–3.44; *p* = 0.07). It has been shown that L858R point mutations are detected more frequently in MIA with completely different tumor characteristics compared to exon 19 deletions (46). However, as only 3 papers were included in this analysis, the results are perhaps not robust enough, which still needs to be corroborated by subsequent relevant studies.

In 2004, the close link between NSCLC and EGFR mutations was identified for the first time, ushering NSCLC treatment into the period of targeted therapy. More than 80% of EGFR mutations are exon 19 deletion mutations and exon 21 L858R point mutations. According to studies, however, patients with rare EGFR mutations have a worse prognosis than those with sensitive mutations. For instance, the EGFR 20 exon insertion mutations causes a spatial site block in the structure of the EGFR protein, reducing the size of the drug-binding pocket and preventing the EGFR-TKI from binding to its target, thereby allowing the EGFR protein to remain active and the oncogenic signal to persist (47, 48). We had planned to set up further research of rare types of EGFR mutations, but unfortunately, the dearth of studies and the scarcity of data on rare mutations prevented us from implementing our plan, which may be taken into account in a future meta-analysis.

This systematic review and meta-analysis has several major advantages. First, we report for the first time that lung adenocarcinomas presenting as MIA exhibit distinct EGFR mutation features. Secondly, our study included a relevant sample size of 1789 patients whose sources included only articles with a low risk of bias and high quality, which provides a more realistic and convincing reflection of the accuracy of the final results. In addition, the sensitivity analysis performed provides evidence that all the results presented are robust. We also assessed publication bias using Egger’s test to ensure that the results are not biased by publication bias. Most importantly, the search strategy developed by our researchers was thorough to ensure that no valuable literature was missed.

This meta-analysis also has several limitations. First of all, the literature included in our study was all from East Asia, which may compromise the accuracy of the analysis of the incidence of EGFR mutations in non-Asian populations. In addition, all included studies were retrospective studies, and there may be selection bias affecting the final overall results. Furthermore, thoracic CT scan parameters, GGO ratio calculation, and EGFR mutations detection methods differed among the included studies, which may increase the heterogeneity among the studies.

## Conclusion

This systematic review and meta-analysis is the most exhaustive and up-to-date examination of the literature concerning the risk factors linked with EGFR mutations. The final results proved that patients with higher age, female gender, and non-smoking individuals would have a relatively higher incidence of EGFR mutations. Also, the risk of EGFR mutations was increased with larger tumor diameter, tumor imaging presentation of mGGO, and tumor pathological findings of MIA or IAC. Importantly, tumors presenting as MIA are more likely to have the incidence of L858R point mutation. This finding is somewhat helpful in predicting and assessing the molecular pathological alterations in stage IA NSCLC. Based on the aforementioned risk factors associated with EGFR mutations, thoracic surgeons can make an initial assessment of the molecular pathology of early-stage lung nodules in order to minimize unnecessary costs to the patient, thereby reducing the patient’s financial burden. It also reduces the rate of underdiagnosis of patients with EGFR mutations, and ensures that patients with EGFR mutations will be able to detect their mutations in a timely manner and receive more rigorous postoperative follow-up.

## Data availability statement

The original contributions presented in the study are included in the article/Supplementary material, further inquiries can be directed to the corresponding authors.

## Author contributions

JQ: Conceptualization, Data curation, Formal analysis, Investigation, Methodology, Project administration, Writing – original draft. ZM: Data curation, Formal analysis, Methodology, Project administration, Supervision, Writing – review & editing. RL: Data curation, Formal analysis, Investigation, Software, Writing – review & editing. CQ: Formal analysis, Investigation, Writing – review & editing. KW: Formal analysis, Software, Writing – review & editing. BL: Data curation, Software, Writing – review & editing. YT: Conceptualization, Project administration, Supervision, Writing – review & editing. HT: Conceptualization, Funding acquisition, Methodology, Project administration, Supervision, Writing – review & editing.

## Footnotes

^1^https://inplasy.com/inplasy-2023-2-0043

## Supplementary material

Supplementary Figure S1 Sensitivity analysis of demographic and partial thoracic CT information of patients with NSCLC between the EGFR positive and negative groups. **(A)** Old age; **(B)** Larger tumor size; **(C)** Non-smoking individuals; **(D)** Female gender. CT, computed tomography; NSCLC, non-small cell lung cancer; EGFR, epidermal growth factor receptor; OR, odds ratio; CI, confidence interval.

Supplementary Figure S2 Sensitivity analysis of partial thoracic CT information and pathological outcomes of patients with NSCLC between the EGFR positive and negative groups. **(A)** mGGO; **(B)** IAC vs. PGL; **(C)** MIA vs. PGL. CT, computed tomography; NSCLC, non-small cell lung cancer; EGFR, epidermal growth factor receptor; mGGO, mixed ground glass opacity; IAC, invasive adenocarcinoma; MIA, minimally invasive adenocarcinoma; PGL, precursor glandular lesions; OR, odds ratio; CI, confidence interval.

Supplementary Figure S3 Sensitivity analysis of the pathological outcomes of patients with NSCLC between the EGFR mutant subtype groups and wild groups. **(A)** IAC vs. PGL (19del); **(B)** IAC vs. PGL (L858R); **(C)** MIA vs. PGL (19del); **(D)** MIA vs. PGL (L858R). NSCLC, non-small cell lung cancer; EGFR, epidermal growth factor receptor; IAC, invasive adenocarcinoma; MIA, minimally invasive adenocarcinoma; PGL, precursor glandular lesions; 19del, 19 deletion mutations; L858R, the L858R point mutations in exon 21 accounts; OR, odds ratio; CI, confidence interval.
