# Supplementary figures and images for: Distinguishing EGFR mutant subtypes in stage IA non-small cell lung cancer using the presence status of ground glass opacity and final histologic classification: a systematic review and meta-analysis
Source: Front Med (Lausanne). 2023 Dec 6;10:1268846. doi: 10.3389/fmed.2023.1268846 (PMC10731050; doi:10.3389/fmed.2023.1268846)

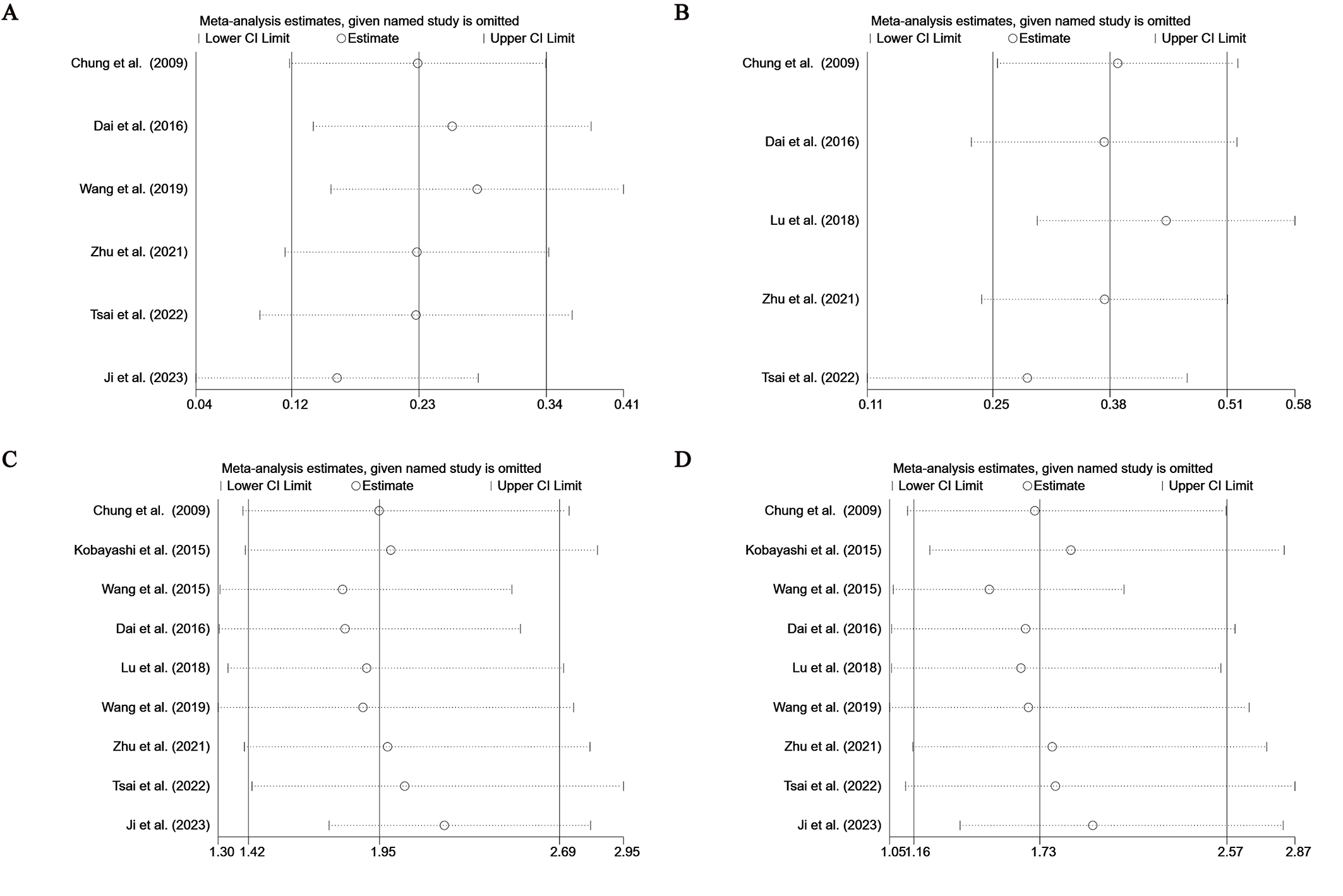

Supplement: Supplementary Figure S1 — Sensitivity analysis of demographic and partial thoracic CT information of patients with NSCLC between the EGFR positive and negative groups. (A) Old age; (B) Larger tumor size; (C) Non-smoking individuals; (D) Female gender. CT, computed tomography; NSCLC, non-small cell lung cancer; EGFR, epidermal growth factor receptor; OR, odds ratio; CI, confidence interval. [file Image_1.TIF]

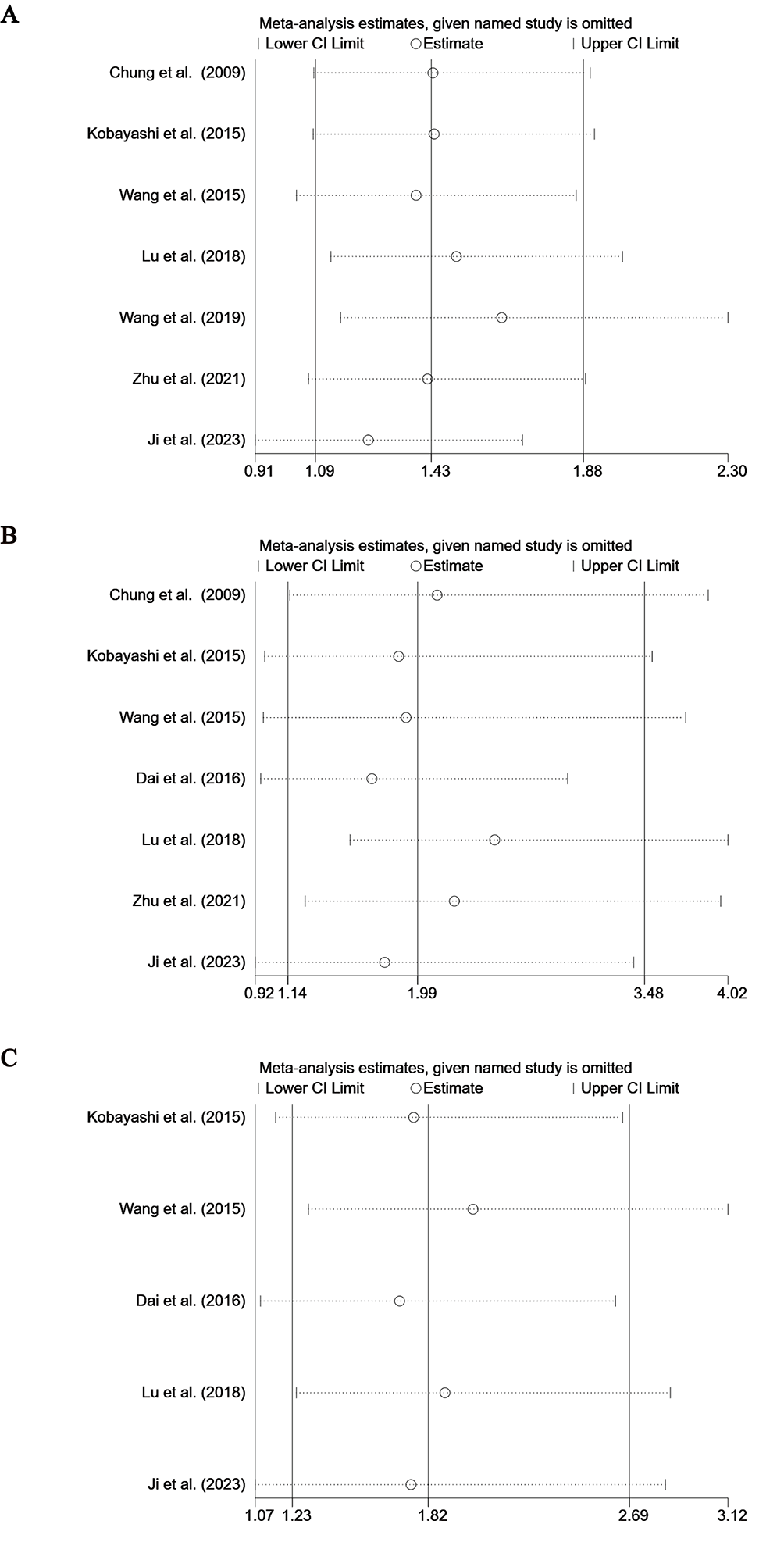

Supplement: Supplementary Figure S2 — Sensitivity analysis of partial thoracic CT information and pathological outcomes of patients with NSCLC between the EGFR positive and negative groups. (A) mGGO; (B) IAC vs. PGL; (C) MIA vs. PGL. CT, computed tomography; NSCLC, non-small cell lung cancer; EGFR, epidermal growth factor receptor; mGGO, mixed ground glass opacity; IAC, invasive adenocarcinoma; MIA, minimally invasive adenocarcinoma; PGL, precursor glandular lesions; OR, odds ratio; CI, confidence interval. [file Image_2.TIF]

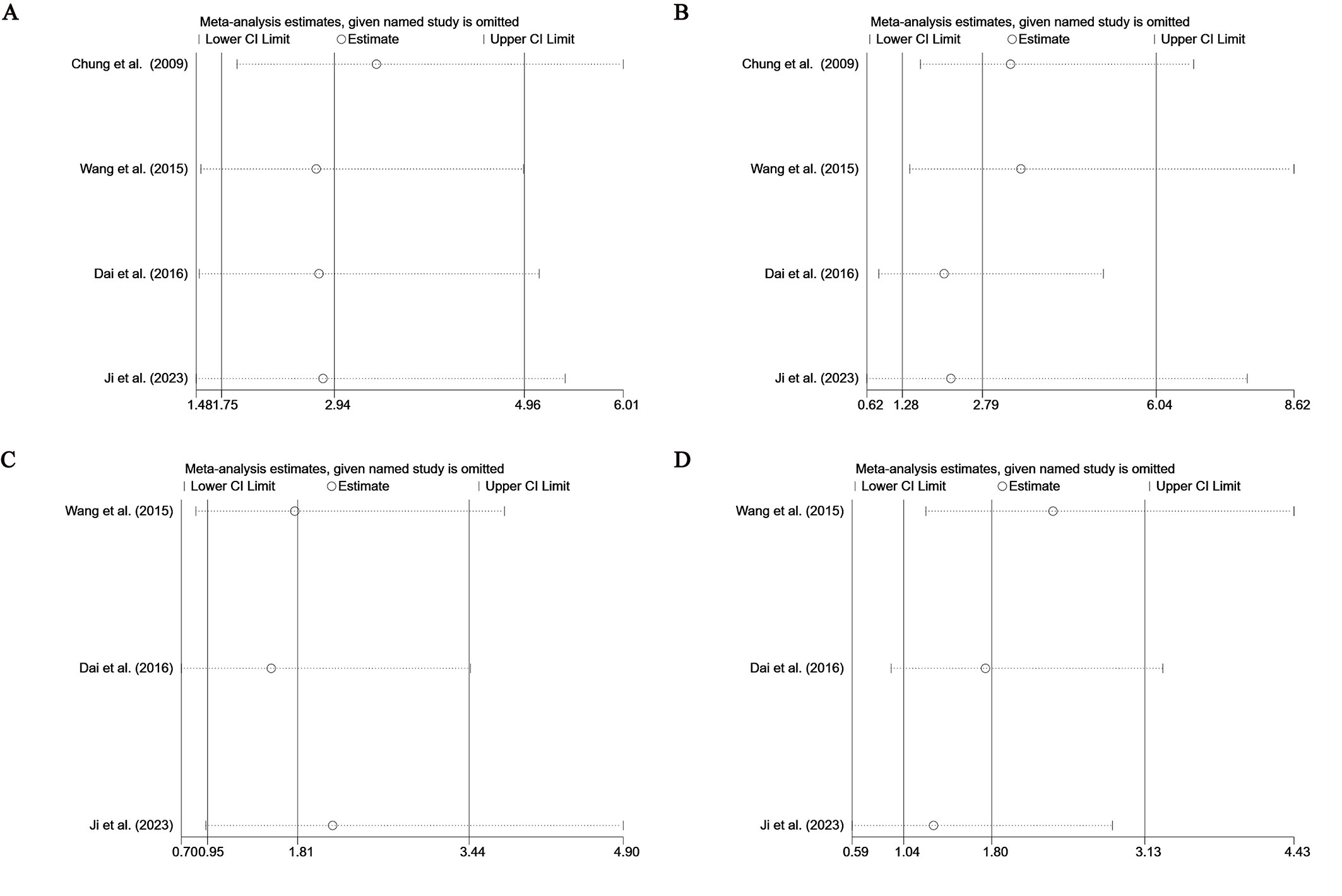

Supplement: Supplementary Figure S3 — Sensitivity analysis of the pathological outcomes of patients with NSCLC between the EGFR mutant subtype groups and wild groups. (A) IAC vs. PGL (19del); (B) IAC vs. PGL (L858R); (C) MIA vs. PGL (19del); (D) MIA vs. PGL (L858R). NSCLC, non-small cell lung cancer; EGFR, epidermal growth factor receptor; IAC, invasive adenocarcinoma; MIA, minimally invasive adenocarcinoma; PGL, precursor glandular lesions; 19del, 19 deletion mutations; L858R, the L858R point mutations in exon 21 accounts; OR, odds ratio; CI, confidence interval. [file Image_3.TIF]
