# Supplementary material for: Distinguishing EGFR mutant subtypes in stage IA non-small cell lung cancer using the presence status of ground glass opacity and final histologic classification: a systematic review and meta-analysis
Source: Front Med (Lausanne). 2023 Dec 6;10:1268846. doi: 10.3389/fmed.2023.1268846 (PMC10731050; doi:10.3389/fmed.2023.1268846)
Supplement: Supplementary file 4 [file Table_1.DOCX]

**Table S1.** Details of search strategy for all databases.

| **Databases** | **Search strategy** |
| --- | --- |
| PubMed | (((((((((((((((multiple primary lung cancer[Title/Abstract]) OR (multiple primary lung cancers[Title/Abstract])) OR (multiple primary lung adenocarcinoma[Title/Abstract])) OR (multiple primary lung adenocarcinomas[Title/Abstract])) OR (synchronous multiple primary lung cancer[Title/Abstract])) OR (metachronous multiple primary lung cancer[Title/Abstract])) OR (synchronous multiple primary lung cancers[Title/Abstract])) OR (metachronous multiple primary lung cancers[Title/Abstract])) OR (("Adenocarcinoma of Lung"[MeSH] OR "lung adenocarcinomas"[Title/Abstract] OR "Adenocarcinoma of Lung"[Title/Abstract] OR "lung adenocarcinoma"[Title/Abstract] OR "adenocarcinoma lung"[Title/Abstract] OR "adenocarcinomas lung"[Title/Abstract]) OR ((((((((((((((((((("Lung Neoplasms"[Mesh]) OR (Lung Neoplasms[Title/Abstract])) OR (Pulmonary Neoplasms[Title/Abstract])) OR (Neoplasms, Lung[Title/Abstract])) OR (Lung Neoplasm[Title/Abstract])) OR (Neoplasm, Lung[Title/Abstract])) OR (Neoplasms, Pulmonary[Title/Abstract])) OR (Neoplasm, Pulmonary[Title/Abstract])) OR (Pulmonary Neoplasm[Title/Abstract])) OR (Lung Cancer[Title/Abstract])) OR (Cancer, Lung[Title/Abstract])) OR (Cancers, Lung[Title/Abstract])) OR (Lung Cancers[Title/Abstract])) OR (Pulmonary Cancer[Title/Abstract])) OR (Cancer, Pulmonary[Title/Abstract])) OR (Pulmonary Cancers[Title/Abstract])) OR (Cancers, Pulmonary[Title/Abstract])) OR (Cancer of the Lung[Title/Abstract])) OR (Cancer of Lung[Title/Abstract])))) OR ((((("carcinoma, non-small-cell lung"[MeSH]) OR (carcinoma, non-small-cell lung[Title/Abstract])) OR (NSCLC[Title/Abstract])) OR (Non-Small-Cell Lung Carcinoma[Title/Abstract])) OR (Non-Small Cell Lung Cancer[Title/Abstract])) OR ((("Neoplasms, Squamous Cell"[Mesh]) OR (Carcinoma, Squamous Cell[Title/Abstract]))) AND ("GGO"[Title/Abstract] OR "GGN"[Title/Abstract] OR "ground glass opacity"[Title/Abstract] OR "ground glass nodule"[Title/Abstract] OR "ground glass nodules"[Title/Abstract] OR "ground glass opacity"[Title/Abstract] OR "ground glass nodule"[Title/Abstract] OR "ground glass nodules"[Title/Abstract] OR "subsolid nodule"[Title/Abstract] OR "subsolid nodules"[Title/Abstract] OR "subsolid pulmonary nodules"[Title/Abstract]) AND (((((((((((((Gene[Title/Abstract]) OR (Cistron[Title/Abstract])) OR (Cistrons[Title/Abstract])) OR (Genetic Materials[Title/Abstract])) OR (Genetic Material[Title/Abstract])) OR (Genetic feature[Title/Abstract])) OR (genetic characteristics[Title/Abstract])) OR (genetic characteristic[Title/Abstract])) OR (genetic features[Title/Abstract])) OR (Genomic alteration[Title/Abstract])) OR (Genomic alterations[Title/Abstract])) OR (EGFR[Title/Abstract])) OR (epidermal growth factor receptor[Title/Abstract]))) |
| Embase | ('Lung Neoplasms' OR 'Pulmonary Neoplasms' OR 'Neoplasms, Lung' OR 'Lung Neoplasm' OR 'Neoplasm, Lung' OR 'Neoplasms, Pulmonary' OR 'Neoplasm, Pulmonary' OR 'Pulmonary Neoplasm' OR 'Lung Cancer' OR 'Cancer, Lung' OR 'Cancers, Lung' OR 'Lung Cancers' OR 'Pulmonary Cancer' OR 'Cancer, Pulmonary' OR 'Cancers, Pulmonary' OR 'Pulmonary Cancers' OR 'Cancer of the Lung' OR 'Cancer of Lung' OR 'Adenocarcinoma of Lung' OR 'Lung Adenocarcinomas' OR 'Lung Adenocarcinoma' OR 'Adenocarcinoma, Lung' OR 'Adenocarcinomas, Lung' OR 'multiple primary lung cancer' OR 'multiple primary lung cancers' OR 'multiple primary lung adenocarcinoma' OR 'multiple primary lung adenocarcinomas' OR 'synchronous multiple primary lung cancer' OR ' carcinoma, non-small-cell lung' OR 'nsclc' OR 'Carcinoma, Squamous Cell':ab,ti OR 'Adenocarcinoma of Lung'/exp) AND ('GGO' OR 'GGN' OR 'ground glass opacity' OR 'ground glass nodule' OR 'ground glass nodules' OR 'ground glass opacity' OR 'ground glass nodule' OR 'ground glass nodules' OR 'subsolid nodule' OR 'subsolid nodules' OR 'subsolid pulmonary nodules':ab,ti OR 'ground glass opacity'/exp) AND ('Gene' OR 'Cistron' OR 'Cistrons' OR 'Genetic Materials' OR 'Genetic Material' OR 'Genetic feature' OR 'genetic characteristics' OR 'genetic characteristic' OR 'genetic features' OR 'Genomic alteration' OR 'Genomic alterations' OR 'EGFR' OR 'epidermal growth factor receptor':ab,ti OR 'epidermal growth factor receptor'/exp) |
| Cochrane Library | ((Lung Neoplasms) OR (Pulmonary Neoplasms) OR (Neoplasms, Lung) OR (Lung Neoplasm) OR (Neoplasm, Lung) OR (Neoplasms, Pulmonary) OR (Neoplasm, Pulmonary) OR (Pulmonary Neoplasm) OR (Lung Cancer) OR (Cancer, Lung) OR (Cancers, Lung) OR (Lung Cancers) OR (Pulmonary Cancer) OR (Cancer, Pulmonary) OR (Cancers, Pulmonary) OR (Pulmonary Cancers) OR (Cancer of the Lung) OR (Cancer of Lung) OR (Adenocarcinoma of Lung) OR (Lung Adenocarcinomas) OR (Lung Adenocarcinoma) OR (Adenocarcinoma, Lung) OR (Adenocarcinomas, Lung) OR (multiple primary lung cancer) OR (multiple primary lung cancers) OR (multiple primary lung adenocarcinoma) OR (multiple primary lung adenocarcinomas) OR (synchronous multiple primary lung cancer) OR (metachronous multiple primary lung cancer) OR (carcinoma, non-small-cell lung) OR (Carcinoma, Squamous cell)) AND ((GGO) OR (GGN) OR (ground glass opacity) OR (ground glass nodule) OR (ground glass nodules) OR (ground glass opacity) OR (ground glass nodule) OR (ground glass nodules) OR (subsolid nodule) OR (subsolid nodules) OR (subsolid pulmonary nodules)) AND ((Gene) OR (Cistron) OR (Cistrons) OR (Genetic Materials) OR (Genetic Material) OR (Genetic feature) OR (genetic characteristics) OR (genetic characteristic) OR (genetic features) OR (Genomic alteration) OR (Genomic alterations) OR (EGFR) OR (epidermal growth factor receptor)) |
| Web of Science | ((Lung Neoplasms) OR (Pulmonary Neoplasms) OR (Neoplasms, Lung) OR (Lung Neoplasm) OR (Neoplasm, Lung) OR (Neoplasms, Pulmonary) OR (Neoplasm, Pulmonary) OR (Pulmonary Neoplasm) OR (Lung Cancer) OR (Cancer, Lung) OR (Cancers, Lung) OR (Lung Cancers) OR (Pulmonary Cancer) OR (Cancer, Pulmonary) OR (Cancers, Pulmonary) OR (Pulmonary Cancers) OR (Cancer of the Lung) OR (Cancer of Lung) OR (Adenocarcinoma of Lung) OR (Lung Adenocarcinomas) OR (Lung Adenocarcinoma) OR (Adenocarcinoma, Lung) OR (Adenocarcinomas, Lung) OR (multiple primary lung cancer) OR (multiple primary lung cancers) OR (multiple primary lung adenocarcinoma) OR (multiple primary lung adenocarcinomas) OR (synchronous multiple primary lung cancer) OR (metachronous multiple primary lung cancer) OR (Carcinoma, Squamous Cell) OR (carcinoma, non-small-cell lung)) AND ((GGO) OR (GGN) OR (ground glass opacity) OR (ground glass nodule) OR (ground glass nodules) OR (ground glass opacity) OR (ground glass nodule) OR (ground glass nodules) OR (subsolid nodule) OR (subsolid nodules) OR (subsolid pulmonary nodules)) AND ((Gene) OR (Cistron) OR (Cistrons) OR (Genetic Materials) OR (Genetic Material) OR (Genetic feature) OR (genetic characteristics) OR (genetic characteristic) OR (genetic features) OR (Genomic alteration) OR (Genomic alterations) OR (EGFR) OR (epidermal growth factor receptor)) |
